# Interferometric Fiber Optic Probe for Measurements of Cavitation Bubble Expansion Velocity and Bubble Oscillation Time

**DOI:** 10.3390/s23020771

**Published:** 2023-01-10

**Authors:** Emil Zubalic, Daniele Vella, Aleš Babnik, Matija Jezeršek

**Affiliations:** Laboratory for Laser Techniques, Faculty of Mechanical Engineering, University of Ljubljana, Aškerčeva Cesta 6, 1000 Ljubljana, Slovenia

**Keywords:** optically induced cavitation, bubble dynamics measurement, fiber optic probe hydrophone, fiber optic probe interferometer, bubble oscillation time, bubble expansion velocity

## Abstract

Cavitation bubbles are used in medicine as a mechanism to generate shock waves. The study of cavitation bubble dynamics plays a crucial role in understanding and utilizing such phenomena for practical applications and purposes. Since the lifetime of cavitation bubbles is in the range of hundreds of microseconds and the radii are in the millimeter range, the observation of bubble dynamics requires complicated and expensive equipment. High-speed cameras or other optical techniques require transparent containers or at least a transparent optical window to access the region. Fiber optic probe tips are commonly used to monitor water pressure, density, and temperature, but no study has used a fiber tip sensor in an interferometric setup to measure cavitation bubble dynamics. We present how a fiber tip sensor system, originally intended as a hydrophone, can be used to track the expansion and contraction of cavitation bubbles. The measurement is based on interference between light reflected from the fiber tip surface and light reflected from the cavitation bubble itself. We used a continuous-wave laser to generate cavitation bubbles and a high-speed camera to validate our measurements. The shock wave resulting from the collapse of a bubble can also be measured with a delay in the order of 1 µs since the probe tip can be placed less than 1 mm away from the origin of the cavitation bubble. By combining the information on the bubble expansion velocity and the time of bubble collapse, the lifetime of a bubble can be estimated. The bubble expansion velocity is measured with a spatial resolution of 488 nm, half the wavelength of the measuring laser. Our results demonstrate an alternative method for monitoring bubble dynamics without the need for expensive equipment. The method is flexible and can be adapted to different environmental conditions, opening up new perspectives in many application areas.

## 1. Introduction

Fiber optic probe hydrophones (FOPHs) are well-known measurement systems [1,2,3,4], commonly used in medicine and in all fields where there is a need to measure fast transient pressure waves or shock waves with high amplitude and broad bandwidth [5]. To generate an optically induced ultrasonic wave, a high-energy pulsed laser and a breakdown of the liquid medium is usually employed, leading to the formation of cavitation bubbles, followed by the emission of a shock wave after the bubble has collapsed [6,7]. This concept has been used in many application fields, such as targeted tissue destruction, cancer therapy [8], intraocular surgery [9], irrigation of endodontic root canals [10], and many other medical applications [11,12,13], due to the high amplitude and frequency of the wave that can be achieved.

In laser-induced phenomena, bubble dynamics plays a crucial role as it determines the characteristics of the generated shockwave waveform [14]. Many experiments and models have been implemented to explain or predict the dynamics, the maximum bubble expansion ratio, or multiple bubble rebounds [15,16,17,18]. Often, a very expensive high-speed camera [19,20,21] and other complex techniques such as shadow photography, high-speed imaging, laser transmission probes, and laser deflection probes have been used to observe bubble evolution in water or a tissue phantom [22,23,24,25,26,27,28] and consequently validate models that predict bubble dynamics and/or pressure shock wave formation.

Most of these studies have been conducted by considering a short laser pulse of known energy as the driving factor for the bubble life cycle. Other studies have shown that similar effects can be achieved by using continuous wave lasers [29,30,31,32] that homogeneously heat the water. Although this approach is semi-stochastic, bubble formation and collapse occur multiple times, with a quasi-constant repetition frequency, depending on the laser power, wavelength, and tip geometry [33].

FOPHs have been used extensively to monitor pressure and temperature changes in fluids. The advantage of such systems is a higher frequency bandwidth that is not limited by the mechanical response of the system, as is the case with PVDF (polyvinylidene fluoride) needle hydrophones [34]. In fact, since a pressure wave alters the optical properties of a liquid, by monitoring the intensity of the back-reflected light, the changes in the refractive index of the liquid can be estimated, and hence so can the pressure variations. The bandwidth is limited only by the electronics used to acquire the signal. Although FOPH sensors are widely used, they remain limited to the measurement of pressure and temperature [35]. Kimura et al. [36] and Horiba et al. [37] have observed distortions in measured signals, linking them to the presence of cavitation bubbles on the fiber tip. In the case of laser-induced bubble formation, there is still a lack of correlation between the bubble dynamics and the FOPH working principle. This could be the target for new-perspective applications.

Here, we present a simple method, using a fiber optic probe hydrophone system to not only measure the pressure shock wave resulting from bubble collapses but also to track the expansion velocity of the bubble wall using an interferometric phenomenon. From the interferometric FOPH signal, we can estimate the lifetime of the cavitation bubble, which in our case is ≈220 µs. To validate the use of a fiber optic probe system for this type of measurement, a high-speed camera with a frame rate of 100,000 fps is used. Compared to other methods, a fiber optic probe has the advantage that it can be used even in opaque containers. The diameter of the probe tip is 125 µm, making it minimally invasive and requiring only a single access point to the region of interest. The system has a temporal resolution in the GHz range, limited only by the electronics used to acquire the signal, and a spatial resolution of half the wavelength of the measuring laser, which in our case is 488 nm.

## 2. Materials and Methods

**Figure 1:**
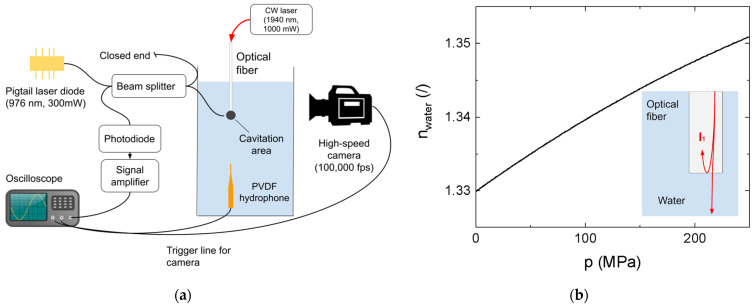
(**a**) Schematic representation of the measuring system; (**b**) water refractive index pressure dependence.

The experimental system is shown schematically in Figure 1a, where the interferometric fiber optic probe and associated subsystems are shown along with two reference measurement systems: a needle hydrophone and a high-speed camera.

*Materials and environment*: A glass water container with dimensions 100×180×250 mm was filled with distilled water and kept at a temperature of 25 °C. To generate cavitation bubbles, we used a continuous-wave laser with a wavelength of 1940 nm and a power of 1000 mW. The light is guided directly into the water via a fiber tip connected directly to the endpiece of a Fotona Skypulse commercial medical laser, which is normally used in endodontics.

*Measuring devices:* The measuring laser is a Thorlabs BL976-PAG500 with a wavelength of 976 nm, spectral bandwidth of 1 nm (−3 dB), and coherence length of 297 μm. The laser was used in continuous-wave mode with a power of 300 mW. The laser diode was operated with the Thorlabs CLD1015 driver and maintained at a temperature of 25 °C. The sensing photodiode is a Thorlabs DET08CFC with a nominal responsivity of 0.667 A/W at the wavelength of 980 nm. The optical fiber tip of the FOPH system was hand-cut from a Thorlabs SM800-5.6-125 single-mode fiber with a diameter of 125 µm and a core diameter of 6 µm. A typical refractive index of the optical fiber core is 1.456. Angled optical connectors of the FC/APC type were used wherever a connection between optical fiber components was necessary (e.g., coupling between the beam splitter and laser diode) to minimize unwanted back reflections.

We used the Standford research SR445 amplifier, which has a bandwidth of 300 MHz with an input impedance of 50 Ω and a nominal gain of 25, in a 2-stage configuration. The measured gain in the 2-stage configuration is 20 for frequencies up to 50 MHz. When performing interferometric measurements, we bypassed the amplifier and connected the diode directly to the oscilloscope.

We used a LeCroy Waverunner oscilloscope with a bandwidth of 500 MHz. When measuring pressure with the FOPH system, we used an integrated low-pass filter with a bandwidth of 20 MHz since this is in a comparable range to the bandwidth of the PVDF hydrophone.

As a reference, we used a PVDF hydrophone from Precision Acoustics with a needle diameter of 0.2 mm. The sensitivity of the hydrophone with the proprietary amplifier is 55 mV/MPa, with a nominal bandwidth of 30 MHz.

A high-speed camera (Photron, Fastcam SA-Z, Tokyo, Japan) was used for reference measurements of vapor bubble dynamics. Images were captured at a frame rate of 100,000 fps and at a resolution of 360 × 384 pixels. Using a macro lens (Sigma, 180 mm F2.8 APO MACRO EX), the optical magnification was 1:1 and the resolution on the object side was 20 μm. A diffused LED background illumination was used. The signal from the PVDF needle hydrophone was used as the trigger point for the system.

*Pressure measurement*: The dependence of the refractive index of water on the wavelength and pressure is described in [38] for pressures ranging from 0 to 250 MPa at an ambient temperature with a variant of the Sellmeier equation (Equation (1)).

(1) $$n_{water}^{2}=1+\sum_{i=1}^{3}\frac{\left(\alpha_{i}p^{2}+\beta_{i}p+\delta_{i}\right)\lambda^{2}}{\lambda^{2}-\left(A_{i}p^{2}+B_{i}p+\Delta_{i}\right)}$$

where $n_{water}$ is the refractive index of water, λ is the wavelength of light, p is the pressure, and $\alpha_{i},\beta_{i},\delta_{i},A_{i},B_{i},$ and $\Delta_{i}$ are coefficients found in the work of Weiss et al. [38]. The reflectance of light at the water–fiber interface is calculated from the Fresnel equation (Equation (2)) for a perpendicular angle of incidence.

(2) $$r={\left(\frac{n_{water}-n_{fiber}}{n_{water}+n_{fiber}}\right)}^{2},$$

where r is the reflectance of the surface and n is the refractive index of the two materials in contact, in our case, water and optical fiber. The measured reflectance, on the other hand, is:

(3) $$r=\frac{P_{ref}}{P_{las}}=\frac{4P_{diode}}{P_{las}}f,$$

where $P_{ref}$ is the reflected power and $P_{las}$ is the measuring laser power. The factor 4 is introduced because the light intensity is halved two times by the beam splitter (Thorlabs TN980R5A2A). The factor f represents the losses inherent to the system and is calculated as the ratio between calculated reflectivity and measured reflectivity. The experiments were performed at a constant ambient temperature of 23 ± 1 °C and humidity of 50 ± 10%. Since they mainly depend on the fiber probe tip surface quality, a cleanly cut probe tip will result in lower losses. Ideally, at 0.1 MPa pressure, the back reflect power at the diode should be 154 µW; we measured 80 µW, giving a loss factor f=0.53.

**Figure 2:**
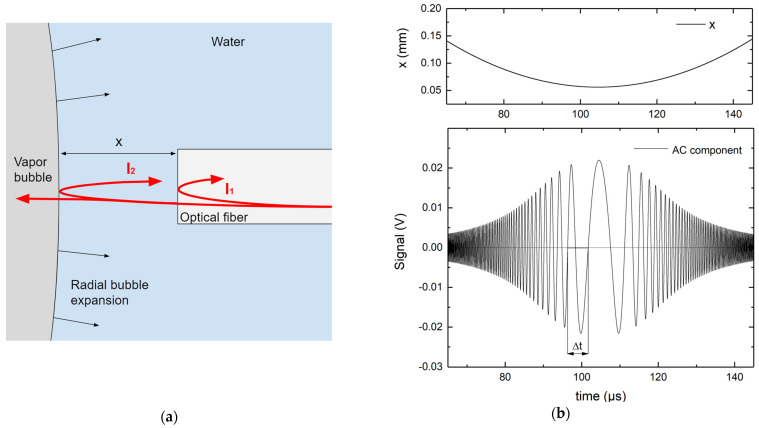
(**a**) Primary and secondary reflection of the laser source when the fiber tip is in close proximity to the bubble wall. (**b**) Top: the bubble wall distance from the fiber probe tip as a function of time that was used for the simulation model. Bottom: expected AC interferometric signal, treating the bubble as a flat mirror.

*Interferometric measurements of the bubble wall expansion velocity*: The oscillations present in the measured signal are related to the expansion of the vapor bubble. When the FOPH probe is sufficiently close to the vapor bubble wall (approximately 0.1 mm), another detectable back reflection occurs at the water–vapor interface (Figure 2a).

The primary reflection occurs at the fiber–water interface and a secondary reflection occurs at the water–vapor interface. The secondary reflected light interferes with the primary reflection in a configuration similar to the Newton interferometer. The difference is that the interferometric fringes appear with changes in time since the bubble surface is not stationary.

Due to beam divergence, a decreasing distance between the fiber tip and the bubble results in increasing signal levels, as depicted in Figure 2b. When the bubble contracts, a specular signal is expected. A simplification of this phenomenon could be described by assuming a Gaussian divergent beam exiting the FOPH probe tip and treating the bubble as a flat mirror. Therefore, the intensity $I_{2}$ of the light beam reflected back into the fiber is proportional to the ratio between the area of the fiber core (constant) and the area of the reflected light beam (which increases with distance). The absorptivity of the light is neglected since it is very small at the used wavelength [39]. The expected signal AC component, shown in Figure 2b, is the result of interference between the primary and secondary reflections and is proportional to the term $I_{AC}$ in Equation (4).

(4) $$I_{B}=I_{DC}+I_{AC}=(I_{1}+I_{2})+2\sqrt{I_{1}I_{2}}\cos\left(4\pi \frac{x}{\lambda }\right)$$

where $I_{B}$ is the total back-reflected light intensity, $I_{DC}$ is the sum of the primary and secondary reflection intensities, $I_{AC}$ is the interferometric term, and λ is the wavelength of the measuring laser source. The distance x from the fiber tip is extracted from the camera images and depicted in the Figure 2b top panel. We show in the Section 3 that this is a close approximation to the real measurement.

To determine the bubble wall velocity, we first subtract the DC component present in the measured signal and then perform a zero-crossing detection algorithm. By measuring the period of an oscillation present in the signal, we calculate the absolute velocity of the bubble wall at that time according to the relationship given in Equation (5).

(5) $$v=\frac{\lambda }{2\Delta t}$$

where v is the absolute velocity of the bubble wall with respect to the fiber tip, and Δt is the period of one oscillation (see Figure 2b). We repeat this for each oscillation present in the signal.

By observing the bubble dynamics of the cavitation bubble with the high-speed camera, we measure the bubble radius at each frame, from which the expansion velocity is calculated. The bubble radius is recorded from the beginning of the expansion to the frame preceding the bubble collapse. A parabolic approximation is fitted on the velocity measurements from the recorded camera data.

## 3. Results and Discussion

**Figure 3:**
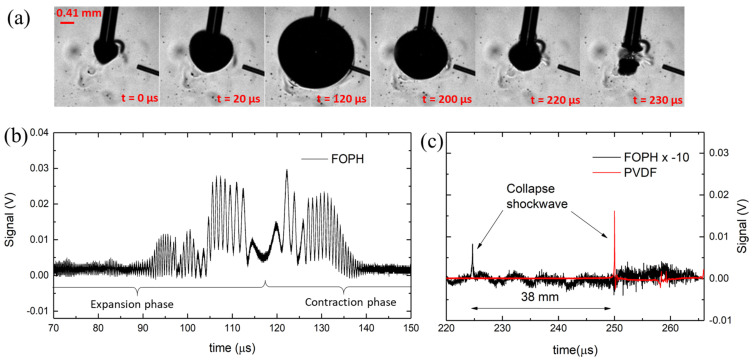
(**a**) Typical bubble dynamics upon laser irradiation through vertically oriented fiber tip; (**b**) single-shot measured signal with FOPH (black line), with visible interferometric fringes at earlier acquisition time; (**c**) average of 50 measured signals with FOPH, where the pressure-induced spike resulting from the shock wave impact with the optical fiber is visible. The signal recorded with the PVDF hydrophone needle is shown in red. In all graphs, the time origin is at the initialization of the bubble, shown in (**a**) at t = 0 μs.

Figure 3a shows the formation of a local bubble upon absorption of laser light in the water near the tip aperture. The light absorption causes the boiling of the liquid and rapid expansion of a nearly spherical vapor bubble. The bubble expands to a maximum radius at t ≈ 116 µs, converting the laser energy into the potential energy of the bubble. The bubble subsequently contracts symmetrically compared to the expansion phase, as shown in [25]. It finally collapses in the time interval t ≈ 220–230 µs. The complete time sequence of the dynamics shown in Figure 3a is common to many other phenomena of laser-induced cavitation in water [16,18,25]. As a result of the collapse, the energy is converted into a shock wave that propagates through the water medium at supersonic speed until it reaches the speed of sound (~1500 m/s), typically after 0.6 mm of travel [7]. A quantitative estimation of the important features involved in the dynamics can be provided by recording the light intensity, which is back-reflected at the FOPH–water interface and converted into an electrical signal. The specification of each single component of the FOPH is known, so we convert a measured electric signal backward to a percentage of reflected light and hence variations of refractive index and absolute pressure (see details in the Section 2).

**Figure 4:**
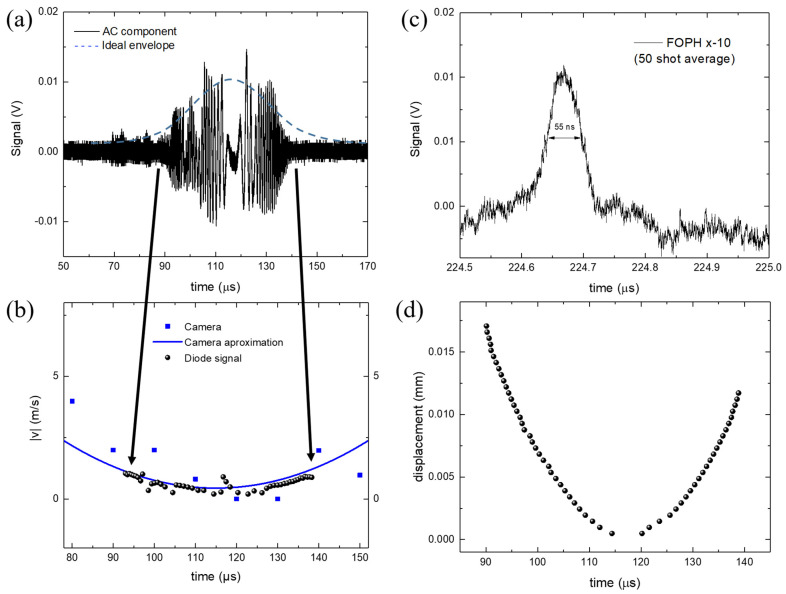
(**a**) AC component of the measured signal; (**b**) bubble wall absolute velocity; (**c**) detail of the bubble collapse shockwave, averaged over 50 measures; (**d**) displacement of the bubble wall relative to the tip position. In all graphs, the time origin is at the initialization of the bubble.

A typical signal recorded by the FOPH is depicted in Figure 3b and an average of over 50 recordings in Figure 3c. In all graphs, the origin of the time axis is at the initialization of the bubble, when it starts to grow. In the first part of the dynamics, temporally before the collapse event, we can observe the interferometric phenomena during the expansion and the contraction of the bubble wall in the proximity of the FOPH. Figure 3b shows that the bubble wall is detected by the interference signal within a time interval of 90–140 μs, where the envelope of the measured signal is given by the total back-reflected light intensity (see Equation (4)). In an ideal case, the amplitude of the signal directly correlates to the distance between the probe tip and bubble wall, but CW lasers cause multiple bubble collapses to form in a short time (Chudnovskii et al. [33] reported a repetition rate of 74 Hz), compromising the homogeneity of the water near the fiber tip, causing rapid jumps in the measured signal. Hence, we extrapolate the bubble wall velocity, as described in the Section 2, without relying on the amplitude measurement. Velocity data points for each increment Δ*t* are displayed in Figure 4b. It is noted that the speed is an absolute value and does not account for the direction change of the bubble wall (expansion and contraction phases). For comparison, the parabolic approximation fit to the data acquired by the camera is shown, revealing a good match with the velocity data points measured with FOPH (see Figure 4b). The bubble wall displacement can be measured by following the same principle since each oscillation corresponds to a displacement of 488 nm from the first observed oscillation. We assume a direction change when v is zero. Figure 4d shows the displacement of the bubble wall relative to the position of the measurement tip (dimension X in Figure 2a). The bubble is detected when it was less than 0.02 mm from the measurement tip. This relatively small measurement range is due to the laser beam divergence. The measurement range can be increased by using a larger-diameter optical fiber, which will reduce the beam divergence and increase the power of the back-reflected light.

The measured signal envelope differs from an expected ideal curve. Debris and smaller bubbles originating from previous bubble collapses can affect the back-reflected light from both the primary and secondary reflections, dragging the envelope away from an ideal signal. In Figure 4a, an approximately symmetric AC signal can be observed during the period between 115 µs and 120 µs, where v ~ 0. We speculate that the smaller peaks in this period can be explained by a superposition effect of surface oscillations caused by turbulence within the bubble. A stationary bubble should result in a DC signal whose amplitude depends on the interferometric condition when the bubble is at the point of maximum expansion. Assuming that the growth and shrinkage of the bubble are symmetric events in time, we can estimate half of the bubble oscillation time as the time between the maximum expansion of the bubble when v=0 and the time when the bubble collapses. Under this assumption, the oscillation time in our case is ~220 µs.

A delay between the detected shock wave and the exact time of bubble collapse will always be present, introducing an error proportional to the distance between the measuring tip and the shock wave source. We minimize this error by placing the fiber optic tip in close proximity to the bubble source (less than 1 mm away), whereas a classical hydrophone would be damaged if placed at the same distance. In our experiment, the needle hydrophone is located 38 mm away from the FOPH. From the measured signal, we can accurately determine the time of bubble collapse, which is at ~224 µs. The distance between the fiber tip and the shock wave source is 0.95 mm, resulting in a time delay between shock wave generation and detection of <0.7 µs, which is negligible for our measurements. The measured shockwave is presented in more detail in Figure 4c.

The peak at time t = 224 µs corresponds to a shock wave peak pressure of 10.8 MPa, calculated by combining Equation (1) and Equation (2) (see Section 2). Compared to the PVDF hydrophones, the FOPHs generally have a lower signal-to-noise ratio. This is because the reflectance at the water fiber surface is very low in the first place (0.2% in ambient conditions) and the changes in reflectance are also very small (−0.03% at 100 MPa), which means a sensitivity of 0.1 mV/MPa for our system. For this reason, the signal presented for a pressure wave measurement is averaged 50 times. Given it has the highest sensitivity, the signal from the PVDF hydrophone is preferred as the trigger input for the camera and the FOPH.

The bubble wall expansion velocity is measured with the camera up to the time of 250 µs and is within the expected range, similar to observations in other studies of cavitation bubbles [14]. The bubble wall velocity can only be measured in a smaller interval with the interferometric setup. On the other hand, a much larger number of measurements can be made in the same time interval, easily increasing the acquisition frequency by tenfold compared to the high-speed camera. In addition, since interference fringes occur at fixed spatial intervals that are multiples of half the wavelength, a faster-moving bubble wall increases the oscillation frequency without affecting the accuracy of the velocity measurement. It is worth noting that the reflectance increases significantly when the fiber tip is completely covered by the vapor bubble. A sudden increase in reflected light intensity, with no interference fringes present, is a strong indication of the presence of vapor as opposed to the water on the fiber probe tip. When the fiber tip’s exact location is determined, via a positioning system, the maximum size of the bubble can also be determined.

In sum, FOPHs can monitor the dynamics of the bubble with a spatial resolution limited only by the laser wavelength (see Equation (5)). This measurement technique can also be used when the fluid container is opaque. Compared to other approaches, FOPH is minimally invasive and capable of operating in turbulent regimes, and no transparent optical window is required, as is the case with measurement techniques such as beam deflection probes [40], beam transmission probes [41], or Mach–Zehnder interferometry [28].

The FOPH can be further improved by using an optical quadrature interferometer configuration, as is demonstrated in [42]. This would increase the resolution and help determine the direction of bubble wall movement.

## 4. Conclusions

We have shown how a fiber optic probe hydrophone can be used to measure the dynamics of optically generated cavitation bubbles in a liquid medium. The velocity of the cavitation bubble wall is determined by measuring the interferometric fringe period, while a shock wave causes a pronounced spike in the measured signal. When measuring the expansion velocity of the bubbles, the spatial resolution remains at 488 nm, half the wavelength of the measuring laser, while the temporal resolution is limited only by the electronics in the GHz range. By combining the information on the bubble wall expansion velocity and the exact time of bubble collapse, the lifetime of cavitation bubbles can be quickly estimated. We validated our measurements with a high-speed camera, finding good agreement between the results obtained with these two approaches. Fiber optic probe hydrophones can be used in environments where other measurement techniques are not applicable, such as narrow channels, hard-to-reach areas, or opaque containers. In principle, a fiber tip probe can also be used for sensing chemical properties, such as a change in pH value [43], and can also be used with fluids other than water to measure both bubble dynamics and pressure shock waves, opening up new technological opportunities. This novel approach to studying bubble dynamics paves the way for low-cost and flexible optical-fiber-based sensing of cavitation phenomena.

## Data Availability

The data are available on request.
